# Association of C-type lectin-like receptor 2 and galectin-1 with portal vein system thrombosis in HBV-related liver cirrhosis

**DOI:** 10.3389/fmed.2023.1228636

**Published:** 2023-08-31

**Authors:** Yiyan Zhang, Xintong Zhang, Xiangbo Xu, Xiaozhong Guo, Shixue Xu, Shaoze Ma, Jihong Chen, Xingshun Qi

**Affiliations:** ^1^Liver Cirrhosis Study Group, Department of Gastroenterology, General Hospital of Northern Theater Command, Shenyang, China; ^2^Postgraduate College, China Medical University, Shenyang, China; ^3^Chinese People’s Liberation Army General Hospital, Chinese People’s Liberation Army Medical School, Beijing, China; ^4^Postgraduate College, Shenyang Pharmaceutical University, Shenyang, China; ^5^Postgraduate College, Dalian Medical University, Dalian, China

**Keywords:** portal venous system thrombosis, C-type lectin-like receptor 2, galectin-1, hepatitis B virus infection, liver cirrhosis

## Abstract

**Background and aims:**

Hepatitis B virus (HBV) infection is the most common cause of liver cirrhosis. Portal venous system thrombosis (PVST) is a major complication of liver cirrhosis. Recently, it has been shown that C-type lectin-like receptor 2 (CLEC-2) and galectin-1 participate in the activation and aggregation of platelets, thereby promoting the development of thrombosis. This cross-sectional study aims to evaluate the association of serum CLEC-2 and galectin-1 levels with PVST in patients with HBV-related liver cirrhosis.

**Methods:**

Overall, 65 patients with HBV-related liver cirrhosis were included, of whom 23 had PVST and 42 did not have. Serum CLEC-2 and galectin-1 levels were measured using enzyme-linked immunosorbent assay kits. PVST was assessed by contrast-enhanced computed tomography and/or magnetic resonance imaging scans. Subgroup analyses were conducted according to the degree and location of PVST.

**Results:**

Patients with PVST had significantly higher serum CLEC-2 (*p* = 0.006) and galectin-1 (*p* = 0.009) levels than those without. Patients with partial/complete PVST or fibrotic cord (*p* = 0.007; *p* = 0.002), but not those with mural PVST (*p* = 0.199; *p* = 0.797), had significantly higher serum CLEC-2 and galectin-1 levels than those without PVST. Patients with superior mesenteric vein thrombosis had significantly higher serum CLEC-2 (*p* = 0.013) and galectin-1 (*p* = 0.025) levels than those without PVST. Patients with main portal vein thrombosis had higher serum CLEC-2 (*p* = 0.020) and galectin-1 (*p* = 0.066) levels than those without PVST, but the difference in serum galectin-1 level was not significant between them.

**Conclusion:**

Serum CLEC-2 and galectin-1 levels may be associated with the presence of PVST in HBV-related cirrhotic patients, but this association should be dependent upon the degree of PVST.

## 1. Introduction

Hepatitis B virus (HBV) infection is the most common etiology of liver cirrhosis (1). Portal venous system thrombosis (PVST) is one of major complications of liver cirrhosis (2). Its prevalence ranges from 1 to 26%, which increases with the severity of liver disease (3, 4). In addition, PVST can increase the risk of long-term death, portal hypertension-related bleeding, ascites, and acute kidney injury in cirrhotic patients (5). Therefore, it is necessary to explore the mechanism regarding the development of PVST in HBV-related liver cirrhosis.

C-type lectin-like receptor 2 (CLEC-2) is a type II transmembrane receptor composed of an extracellular ligand-binding C-type lectin-like domain, stalk region, single transmembrane helix, and short cytoplasmic tail (6). It is encoded by the gene CLEC1B located on 12p13.31 with a molecular weight of 32-40 kDa (7). It is highly expressed in megakaryocytes and platelets and slightly expressed in myeloid cell subsets (8, 9). It has been shown that CLEC-2 mediates the activation and aggregation of platelets (10). Moreover, deep venous thrombosis is significantly inhibited in CLEC-2-deficient mice, suggesting that CLEC-2, especially platelet CLEC-2, should be critical for the development of venous thrombosis (11).

Galectin-1 is the firstly discovered member of the galectin family and belongs to the prototype galectin containing a carbohydrate-binding domain, which can exist as monomers or dimers (12). It is a 14.5 kDa protein encoded by the gene LSGALS1 located on 22q12 (13). Due to the lack of signal peptides, galectin-1 is secreted through a non-classical secretion pathway that translocate directly across the plasma membrane, which performs important biological functions both inside and outside cells (14). Galectin-1 knockout mice have a significantly longer median tail bleeding time than wild-type mice (15). Furthermore, galectin-1-deficient platelets exhibit impairment in fibrinogen adhesion and clot retraction, suggesting that galectin-1 should contribute to hemostasis and involve in thrombosis (15).

Some animal studies have demonstrated a relationship of CLEC-2 and galectin-1 with venous thrombosis, but human studies are lacking (10, 15–17). On the other hand, their association with PVST has never been explored yet. For this reason, this cross-sectional study aimed to evaluate the relationship between CLEC-2/galectin-1 and PVST in patients with HBV-related liver cirrhosis, and further explore the impact of degree and location of PVST on their relationship.

## 2. Materials and methods

### 2.1. Study design

This study followed the Declaration of Helsinki and was approved by the Medical Ethical Committee of the General Hospital of Northern Theater Command (approval number: Y2023-008). All patients who were admitted to the Department of Gastroenterology of the General Hospital of Northern Theater Command between January 2020 and August 2022 were selected. Inclusion criteria were as follows: (1) patients were diagnosed with HBV-related liver cirrhosis; (2) patients underwent contrast-enhanced computed tomography (CT) and/or magnetic resonance imaging (MRI); and (3) patients had already agreed to donate their blood samples and their blood samples remained. Exclusion criteria were as follows: (1) patients with a diagnosis of malignancy or history of splenectomy, splenic artery embolization, transjugular intrahepatic portosystemic shunt, or liver transplantation, which are well-known local risk factors of PVST; (2) patients with other thrombotic diseases; (3) patients with coronary heart disease (18) or acute ischemic stroke (19), which may affect serum CLEC-2/galectin-1 level; (4) patients took anticoagulants and antiplatelet drugs within one month prior to their admissions; and (5) PVST could not be accurately determined. Repeated admissions were not excluded.

### 2.2. Diagnosis and definition

Diagnosis of liver cirrhosis was based on medical history, clinical manifestations, cirrhosis-related complications, laboratory tests, imaging, and histology. HBV-related liver cirrhosis would be defined, if cirrhotic patients were diagnosed with HBV infection or had positive hepatitis B surface antigens. Based on the contrast-enhanced CT/MRI imaging, PVST was defined as thrombosis within portal venous system vessels, including left portal vein, right portal vein, main portal vein (MPV), superior mesenteric vein (SMV), splenic vein (SV), or the confluence of SMV and SV (20). Based on the most severe thrombosis in any vessel of the portal venous system, the degree of thrombosis was divided into mural thrombosis (<50%), partial thrombosis (50–80%), complete thrombosis (>80%), and fibrotic cord (20).

### 2.3. Clinical data collection

Demographics, laboratory tests (i.e., white blood cell, platelet, hemoglobin, total bilirubin, aspartate aminotransferase, albumin, and international normalized ratio), and major complications of liver cirrhosis (i.e., acute gastrointestinal bleeding, ascites, and hepatic encephalopathy) at admission were collected. Child-Pugh score and model for end-stage liver disease (MELD) score at admission were calculated (21). Data accuracy was checked by two researchers (XX and JC).

### 2.4. Measurement of CLEC-2 and galectin-1

Blood samples used in the present study were obtained from the remaining samples of cirrhotic patients prospectively collected by our group. After fasting for 12 h, venous blood samples were collected by gel-procoagulant tubes from cirrhotic patients and centrifuged at 3000 rpm for 10 min at room temperature. The supernatant was carefully collected to obtain serum and stored at a −80°C refrigerator until further analyses. Serum CLEC-2 (MM-2468H1, Meimian, Shanghai, China) and galectin-1 (MM-51147H1, Meimian, Shanghai, China) levels were measured using enzyme-linked immunosorbent assay kits according to the manufacturer’s instructions.

### 2.5. Statistical analyses

Continuous variables were expressed as median (interquartile range, [IQR]) and mean ± standard deviation, and their differences between groups were compared with the Mann–Whitney U test (if non-normal distribution) or T-test (if normal distribution). Categorical variables were expressed as frequency (percentage), and their differences between groups were compared with the Chi-square test or Fisher’s exact test. Subgroup analyses were conducted according to the degree and location of PVST. A two-tailed *p* < 0.05 was considered statistically significant. SPSS version 20.0 (IBM, Armonk, New York, United States) and GraphPad Prism version 8.0.1 (GraphPad software Inc., San Diego, California, USA) were used for all statistical analyses.

## 3. Results

### 3.1. Patients’ characteristics

**Figure 1:**
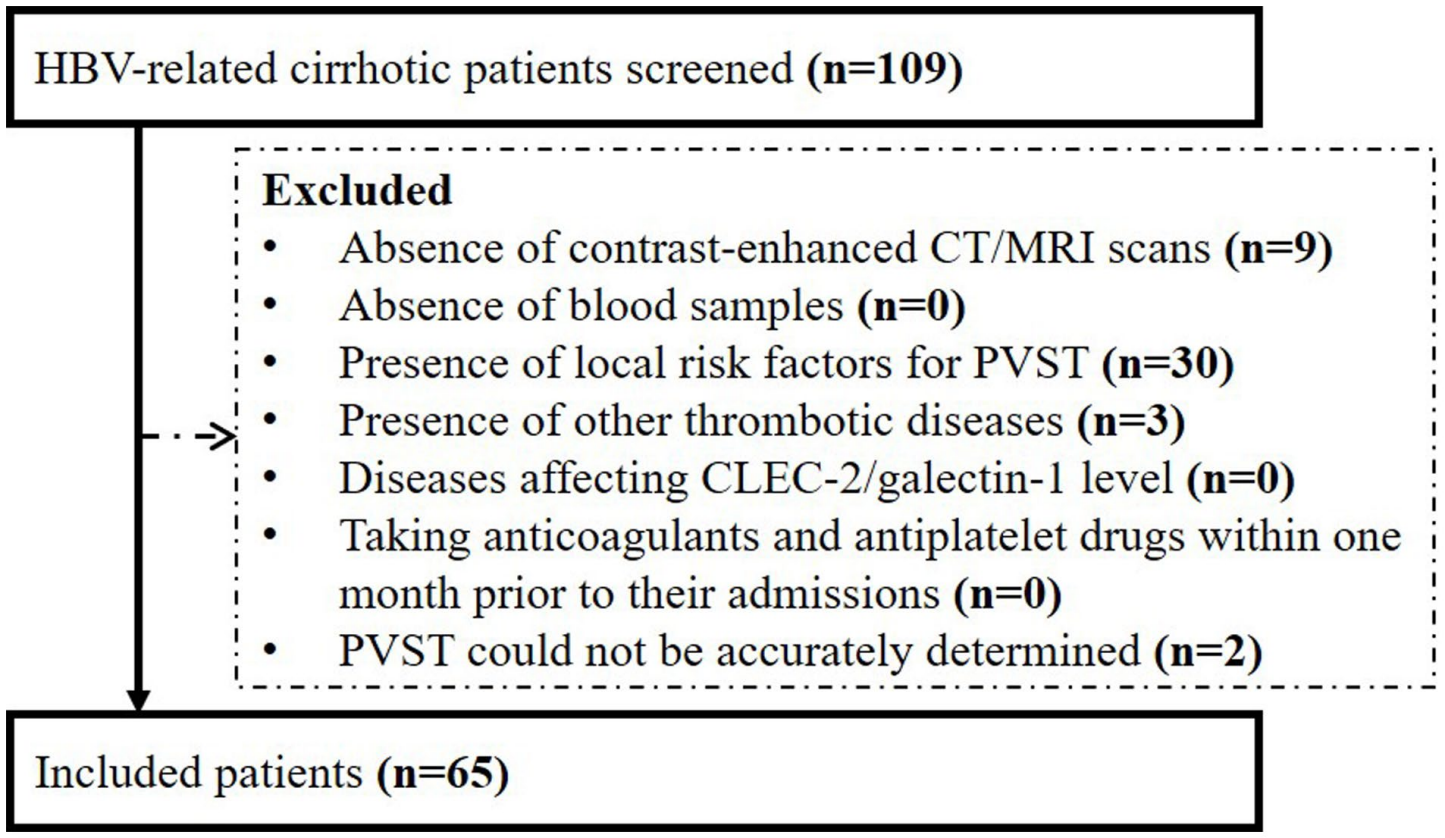
Flow chart of patients’ selection. HBV, hepatitis B virus; CT, computed tomography; MRI, magnetic resonance imaging; PVST, portal venous system thrombosis; CLEC-2, C-type lectin-like receptor 2.

**Figure 2:**
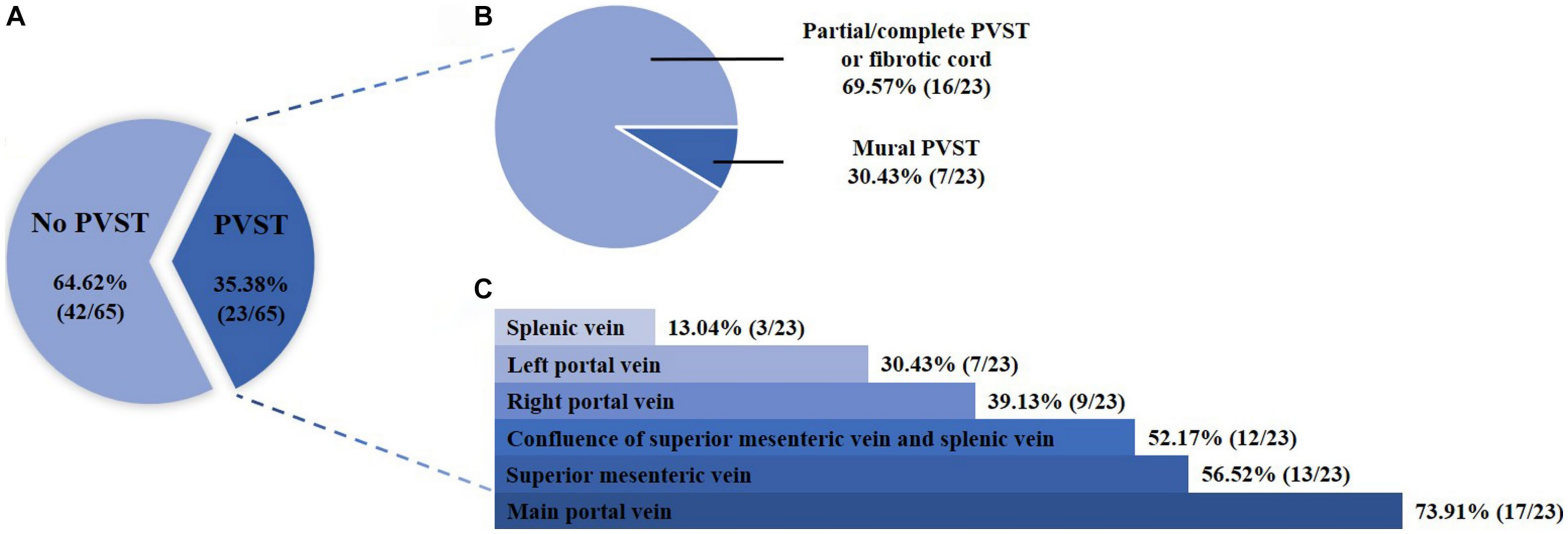
Proportion of patients according to the presence **(A)**, degree **(B)**, and location **(C)** of PVST. PVST, portal venous system thrombosis.

Overall, 65 patients were included (Figure 1). Their median age was 53.30 years (IQR: 46.33–61.54) and 57 (87.69%) patients were male. The median Child-Pugh score and MELD score were 6.00 (IQR: 5.25–8.00) and 10.43 (IQR: 9.07–13.32), respectively (Table 1). Twenty-three patients (35.38%) had PVST (Figure 2A). Among patients with PVST, seven (30.43%) had mural PVST and 16 (69.57%) had partial/complete PVST or fibrotic cord (Figure 2B). The most common location of PVST was MPV (17/23, 73.91%), followed by SMV (13/23, 56.52%) (Figure 2C).

**Table 1:** Patients’ characteristics.

Variables	No. Pts.	Median (IOR), mean ± SD, or frequency (%)
*Demographics*		
Age (years)	65	53.30 (46.33–61.54) 54.19 ± 10.05
Male	65	57 (87.69%)
*Complications of liver cirrhosis*		
Acute gastrointestinal bleeding	65	8 (12.31%)
Ascites	65	36 (55.38%)
Hepatic encephalopathy	65	4 (6.15%)
Portal vein system thrombosis	65	23 (35.38%)
*Laboratory tests*		
White blood cell (10^9^/L)	65	2.80 (2.00–4.20) 3.08 ± 1.44
Platelet (10^9^/L)	65	66.00 (50.50–87.00) 69.91 ± 29.56
Hemoglobin (g/L)	65	111.00 (82.50–133.50) 108.89 ± 33.81
Total bilirubin (μmol/L)	65	18.20 (13.45–31.55) 25.91 ± 17.40
Aspartate aminotransferase (U/L)	65	27.30 (21.81–49.44) 63.88 ± 147.15
Albumin (g/L)	65	34.30 (29.55–38.55) 34.19 ± 6.06
International normalized ratio	64	1.32 (1.20–1.51) 1.36 ± 0.22
MELD score	64	10.43 (9.07–13.32) 11.33 ± 3.20
Child-Pugh score	64	6.00 (5.25–8.00) 7.03 ± 1.94
Child-Pugh class A/B/C	64	33 (51.56%)/21(32.81%)/10(15.63%)
CLEC-2 (pg/mL)	65	622.89 (520.41–970.73) 852.93 ± 565.70
Galectin-1 (pg/mL)	65	38.12 (26.40–131.82) 76.98 ± 65.58

No. Pts, number of patients; IQR, interquartile range; SD, standard deviation; MELD, model for end-stage liver disease; CLEC-2, C-type lectin-like receptor 2.

### 3.2. Difference according to the presence of PVST

**Figure 3:**
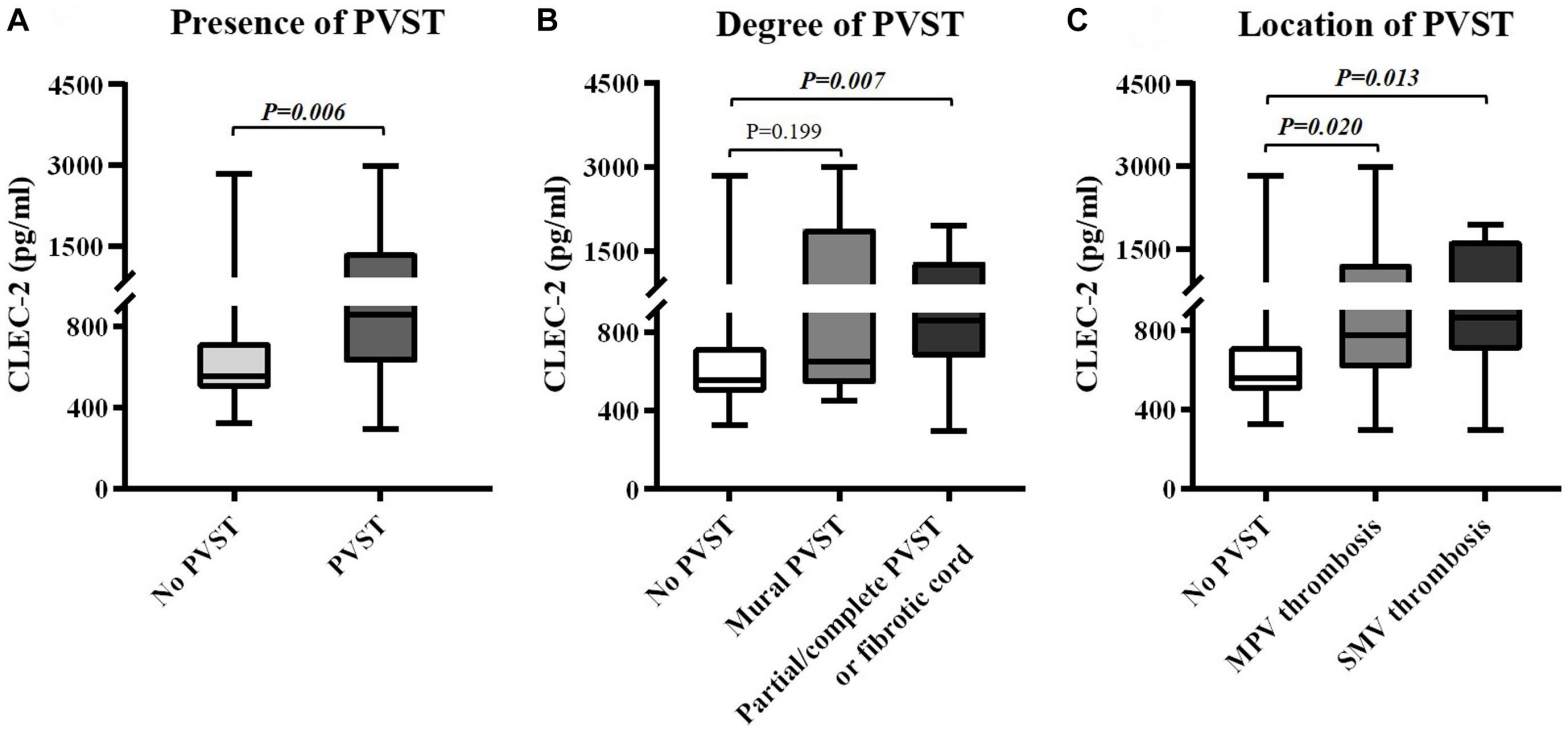
Differences in serum CLEC-2 levels between groups. **(A)** Differences in serum CLEC-2 levels between patients with versus without PVST. **(B)** Differences in serum CLEC-2 levels between patients with mural PVST and patients with partial/complete PVST or fibrotic cord versus those without PVST. **(C)** Differences in serum CLEC-2 levels between patients with MPV thrombosis and patients with SMV thrombosis versus those without PVST. CLEC-2, C-type lectin-like receptor 2; PVST, portal venous system thrombosis; MPV, main portal vein; SMV, superior mesenteric vein.

**Figure 4:**
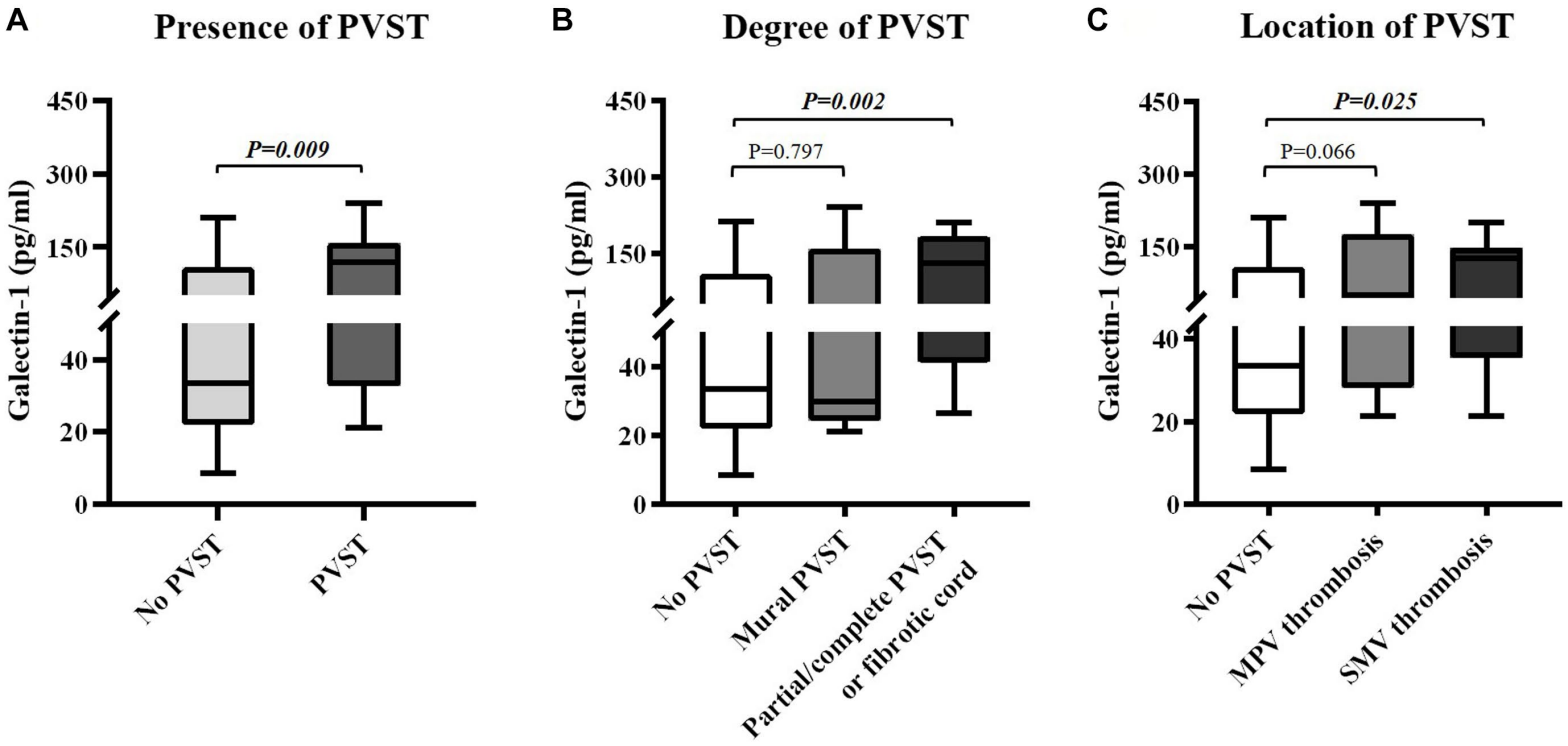
Differences in serum galectin-1 levels between groups. **(A)** Differences in serum galectin-1 levels between patients with versus without PVST. **(B)** Differences in serum galectin-1 levels between patients with mural PVST and patients with partial/complete PVST or fibrotic cord versus those without PVST. **(C)** Differences in serum galectin-1 levels between patients with MPV thrombosis and patients with SMV thrombosis versus those without PVST. PVST, portal venous system thrombosis; MPV, main portal vein; SMV, superior mesenteric vein.

Child-Pugh score (6.96 ± 1.49 vs. 7.07 ± 2.17, *p* = 0.612) and MELD score (11.27 ± 2.36 vs. 11.37 ± 3.62, *p* = 0.900) were statistically similar between patients with and without PVST (Table 2). Patients with PVST had significantly higher serum CLEC-2 (1035.17 ± 630.93 pg/mL vs. 753.14 ± 507.06 pg/mL, *p* = 0.006; Figure 3A) and galectin-1 (104.42 ± 73.37 pg/mL vs. 61.95 ± 56.32 pg/mL, *p* = 0.009; Figure 4A) levels than those without.

**Table 2:** Comparison between patients with versus without PVST.

Variables	No PVST		PVST		*p*-value
	No. Pts.	Median (IQR), mean ± SD, or frequency (%)	No. Pts.	Median (IQR), mean ± SD, or frequency (%)	
*Demographics*					
Age (years)	42	52.23 (43.41–61.47) 53.04 ± 10.35	23	55.16 (50.58–64.54) 56.27 ± 9.33	0.218
Male	42	37 (88.10%)	23	20 (86.96%)	1.000
Child-Pugh score	41	6.00 (5.00–9.00) 7.07 ± 2.17	23	7.00 (6.00–8.00) 6.96 ± 1.49	0.612
MELD score	41	10.03 (8.89–14.66) 11.37 ± 3.62	23	10.88 (9.81–13.22) 11.27 ± 2.36	0.900

No. Pts, number of patients; IQR, interquartile range; SD, standard deviation; PVST, portal vein system thrombosis; MELD, model for end-stage liver disease.

### 3.3. Difference according to the degree of PVST

Child-Pugh score (6.71 ± 1.38 vs. 7.07 ± 2.17, *p* = 0.940) and MELD score (10.76 ± 3.24 vs. 11.37 ± 3.62, *p* = 0.681) were statistically similar between patients with mural PVST and those without PVST (Table 3). Serum CLEC-2 (1147.65 ± 951.76 pg/mL vs. 753.14 ± 507.06 pg/mL, *p* = 0.199; Figure 3B) and galectin-1 (76.83 ± 87.03 pg/mL vs. 61.95 ± 56.32 pg/mL, *p* = 0.797; Figure 4B) levels were not significantly different between patients with mural PVST and those without PVST.

**Table 3:** Comparison between patients with mural PVST and those with partial/complete PVST or fibrotic cord versus those without PVST.

Variables	No PVST		Mural PVST		*p*-value	Partial/complete PVST or fibrotic cord		*p*-value
	No. Pts.	Median (IQR), mean ± SD, or frequency (%)	No. Pts.	Median (IQR), mean ± SD, or frequency (%)		No. Pts.	Median (IQR), mean ± SD, or frequency (%)	
*Demographics*								
Age (years)	42	52.23 (43.41–61.47) 53.04 ± 10.35	7	58.95 (54.57–60.99) 59.29 ± 5.30	***0.027***	16	52.97 (46.33–66.19) 54.95 ± 10.51	0.534
Male	42	37 (88.10%)	7	5 (71.43%)	0.258	16	15 (93.75%)	1.000
Child-Pugh score	41	6.00 (5.00–9.00) 7.07 ± 2.17	7	7.00 (5.00–8.00) 6.71 ± 1.38	0.940	16	7.00 (6.00–7.75) 7.06 ± 1.57	0.490
MELD score	41	10.03 (8.89–14.66) 11.37 ± 3.62	7	10.34 (8.34–10.88) 10.76 ± 3.24	0.681	16	11.09 (9.99–13.31) 11.50 ± 1.94	0.864

No. Pts, number of patients; IQR, interquartile range; SD, standard deviation; PVST, portal vein system thrombosis; MELD, model for end-stage liver disease. Bold italic value indicates results are statistically significant.

Child-Pugh score (7.06 ± 1.57 vs. 7.07 ± 2.17, *p* = 0.490) and MELD score (11.50 ± 1.94 vs. 11.37 ± 3.62, *p* = 0.864) were statistically similar between patients with partial/complete PVST or fibrotic cord and those without PVST (Table 3). Patients with partial/complete PVST or fibrotic cord had significantly higher serum CLEC-2 (985.96±461.53 pg/mL vs. 753.14±507.06 pg/mL, *p* = 0.007; Figure 3B) and galectin 1 116.49±65.98 pg/mL vs. 61.95±56.32 pg/mL, *p* =0.002; Figure 4B) levels than those without PVST.

### 3.4. Difference according to the location of PVST

Child-Pugh score (6.88 ± 1.62 vs. 7.07 ± 2.17, *p* = 0.834) and MELD score (10.85 ± 2.37 vs. 11.37 ± 3.62, *p* = 0.522) were statistically similar between patients with MPV thrombosis and those without PVST (Table 4). Patients with MPV thrombosis had higher serum CLEC-2 (1012.55 ± 674.39 pg/mL vs. 753.14 ± 507.06 pg/mL, *p* = 0.020; Figure 3C) and galectin-1 (96.67 ± 79.43 pg/mL vs. 61.95 ± 56.32 pg/mL, *p* = 0.066; Figure 4C) levels than those without PVST. The difference in serum CLEC-2 level was statistically significant between them, but not serum galectin-1 level.

**Table 4:** Comparison between patients with MPV thrombosis and those with SMV thrombosis versus those without PVST.

Variables	No PVST		MPV thrombosis		*p-*value	SMV thrombosis		*p*-value
	No. Pts.	Median (IQR), mean ± SD, or frequency (%)	No. Pts.	Median (IQR), mean ± SD, or frequency (%)		No. Pts.	Median (IQR), mean ± SD, or frequency (%)	
*Demographics*								
Age (years)	42	52.23 (43.41–61.47) 53.04±10.35	17	58.95 (52.97–67.41) 59.17±8.82	***0.036***	13	57.08 (45.57-62.77) 54.66±10.75	0.627
Male	42	37 (88.10%)	17	15 (88.24%)	1.000	13	12 (92.31%)	1.000
Child-Pugh score	41	6.00 (5.00–9.00) 7.07±2.17	17	7.00 (5.50–8.00) 6.88±1.62	0.834	13	6.00 (5.50-7.00) 6.38±1.12	0.708
MELD score	41	10.03 (8.89–14.66) 11.37±3.62	17	10.38 (9.25–11.34) 10.85±2.37	0.522	13	10.35 (8.76-12.11) 10.67±2.25	0.416

No. Pts, number of patients; IQR, interquartile range; SD, standard deviation; PVST, portal vein system thrombosis; MPV, main portal vein; SMV, superior mesenteric vein; MELD, model for end-stage liver disease. Bold italic value indicates results are statistically significant.

Child-Pugh score (6.38 ± 1.12 vs. 7.07 ± 2.17, *p* = 0.708) and MELD score (10.67 ± 2.25 vs. 11.37 ± 3.62, *p* = 0.416) were statistically similar between patients with SMV thrombosis and those without PVST (Table 4). Patients with SMV thrombosis had significantly higher serum CLEC-2 (1084.65 ± 558.48 pg/mL vs. 753.14 ± 507.06 pg/mL, *p* = 0.013; Figure 3C) and galectin-1 (98.64 ± 61.17 pg/mL vs. 61.95 ± 56.32 pg/mL, *p* = 0.025; Figure 4C) levels than those without PVST.

## 4. Discussion

Some major findings of this study were as follows: (1) serum CLEC-2/galectin-1 level was positively associated with the presence of PVST in HBV-related cirrhotic patients; (2) patients with partial/complete PVST or fibrotic cord, but not those with mural PVST, had significantly higher serum CLEC-2 and galectin-1 levels than those without PVST; (3) patients with MPV or SMV thrombosis had a significantly higher serum CLEC-2 level than those without PVST; and (4) patients with SMV thrombosis, but not those with MPV thrombosis, had a significantly higher serum galectin-1 level than those without PVST.

Platelets are denucleated blood cells from megakaryocytes in the bone marrow (22). Thrombocytopenia, a common complication of liver cirrhosis, is mainly due to decreased synthesis of thrombopoietin in the liver and increased breakdown of platelets in the spleen (23). However, the coagulation system can be often compensated in people with liver disease (24). A reduction in the number of platelets can induce an increase in von Willebrand factor and a decrease in plasma metalloproteinase ADAMTS13, compensatively promoting platelets function (24). On the other hand, platelets adhesion, dissemination, and aggregation are essential for venous thrombosis (25). They also express phosphatidylserine, which provides procoagulant surface for prothrombinase complexes to activate coagulation cascade, and exert pro-inflammatory effects by inducing neutrophil extracellular trap formation, enhancing leukocyte recruitment, and secreting granular contents to activate coagulation system, which further promote the development of venous thrombosis (26, 27). Taken together, it should be reasonable to speculate that the change of platelets function in cirrhotic patients contributes to the development of PVST.

CLEC-2 is an important platelet-activating receptor (9). Its ligand, podoplanin, expresses near the lumen side of endothelial cell in the vascular wall, and the expression of podoplanin is increased in the presence of inflammation and blood flow obstruction (11). Systemic and local inflammation caused by viral hepatitis and increased portal pressure can damage vascular endothelial cells, allowing circulating platelet CLEC-2 to interact with subendothelial podoplanin and possibly with some other unidentified ligands (11, 28, 29). CLEC-2 binds to its ligand to induce phosphorylation of tyrosine residues in a single YXXL motif in the CLEC-2 intracellular domain (30, 31). Splenic tyrosine kinase is activated by binding its tandem Src homologous 2 domain to two tyrosine-phosphorylated YXXL motifs, subsequently triggering downstream signaling pathways that ultimately promote platelets activation and aggregation (7, 17, 32).

Extracellular galectin-1 can induce platelets activation by binding to different surface receptors on platelets (15). As potent platelets agonist, it activates the “inside-out” signal transduction pathway to promote a conformational change in the integrin α_IIb_β_3_ on the platelets surface, changing from a low-affinity/resting state to a high-affinity/active state, exposing the high-affinity binding sites of fibrinogen (15, 33–35). At the same time, galectin-1 can also directly bind to platelets surface integrin α_IIb_β_3_ through α_IIb_ subunit, trigger the “outside-in” signal transduction pathway, and then promote platelets activation and aggregation (15, 34). Moreover, platelets can also express galectin-1, and galectin-1 contained in platelets may play a role in platelets activation, which requires further studies (15, 35).

Our study has for the first time demonstrated a relationship between CLEC-2/galectin-1 level and PVST in cirrhotic patients, which may be dependent upon the degree of PVST. Similarly, an animal study also showed that the progression to stable large thrombosis in CLEC-2-deficient mice was almost completely eliminated compared to the control group, suggesting the critical role of CLEC-2 in pathologically occlusive thrombosis (10). At present, both the Chinese consensus (5) and Baveno VII consensus (36) indicate that the timing of anticoagulation should be related to the degree of PVST. Therefore, both CLEC-2 and galectin-1 levels may be valuable for deciding the initiation of anticoagulant therapy.

Our study also demonstrated that serum galectin-1 level was higher in patients with MPV thrombosis than those without PVST, but this difference was not statistically significant. There were two possible explanations for this unexpected phenomenon. First, Xu et al. (37) showed that galectin-1 level in the peripheral blood of mice decreased with its age. Indeed, the mean age was significantly higher in our patients with MPV thrombosis than those without PVST, suggesting that age might weaken the difference in serum galectin-1 level between the two groups. Second, the sample size of this study was relatively insufficient, thereby compromising the statistical power.

There were some limitations in our study. First, the number of patients included was limited, thus large-scale studies are needed to further verify our results. Second, only patients with HBV-related liver cirrhosis were selected, thus further validation is needed in liver cirrhosis secondary to other etiology.

## 5. Conclusion

Our current findings support a relationship between CLEC-2/galectin-1 and PVST in patients with HBV-related liver cirrhosis. Thus, CLEC-2/galectin-1 may predict the risk of more severe PVST, and they should be considered as potential targets for the prevention and treatment of PVST. In future, the mechanism regarding how CLEC-2/galectin-1 influences the development of PVST needs to be explored.

## Data availability statement

The raw data supporting the conclusions of this article will be made available by the authors, without undue reservation.

## Ethics statement

This study followed the Declaration of Helsinki and was approved by the Medical Ethical Committee of the General Hospital of Northern Theater Command (approval number: Y2023-008).

## Author contributions

XQ: conception and design, administrative support. YZ, XX, SX, JC, and XQ: provision of study materials or patients. YZ, XX, SX, and JC: collection and assembly of data. YZ and XQ: data analysis and interpretation. YZ, XZ, XX, XG, SX, SM, JC, and XQ: manuscript writing. YZ, XZ, XX, XG, SX, SM, JC, and XQ: final approval of manuscript. All authors contributed to the article and approved the submitted version.

## Abbreviations

HBV: hepatitis B virus
PVST: portal venous system thrombosis
CLEC-2: C-type lectin-like receptor 2
CT: computed tomography
MRI: magnetic resonance imaging
MPV: main portal vein
SMV: superior mesenteric vein
SV: splenic vein
MELD: model for end-stage liver disease
IQR: interquartile range
